# Can agricultural mechanization services narrow the income gap in rural China?

**DOI:** 10.1016/j.heliyon.2023.e13367

**Published:** 2023-02-01

**Authors:** Xiance Sang, Xiaofeng Luo, Amar Razzaq, Yanzhong Huang, Sahar Erfanian

**Affiliations:** a College of Economics and Management, Huazhong Agricultural University, Wuhan, China; b Business School, Huanggang Normal University, Huanggang, China; c College of Law and Business, Wuhan Institute of Technology University, Wuhan, China

**Keywords:** Agricultural mechanization services, Rural household income, Income gap, Recentered influence function, China

## Abstract

This paper examines the impact of agricultural mechanization services (AMS) on rural household income and income gap, utilizing the recentered influence function regression method and publicly available data collected through the China Labor-force Dynamics Survey. The results of this study shed light on various impacts of AMS. First, agricultural mechanization services can significantly increase rural household income, but there is heterogeneity in the impact on rural household income in different quantiles. The effect of income growth on medium-income and low-income groups is greater. Second, agricultural mechanization services help to narrow the income gap between rural households and alleviate income inequality in rural areas. Third, the effect of agricultural mechanization services on reducing the income gap between rural households in the eastern and western regions is significantly stronger than that in the central region. Finally, further analysis based on income source structure reveals that agricultural mechanization services can significantly reduce the non-agricultural income gap of rural households, but the impact on the agricultural income gap is negligible. Our findings highlight the importance of government's efforts in promoting the development of agricultural mechanization service market in order to improve the income inequality in rural areas.

## 1 Introduction

With the realization of the goal of building a moderately prosperous society, China's social and economic development has entered a new era, and common prosperity has become an important goal for the country in the new development stage [1]. To narrow the income gap, the Chinese government has made a series of top-level designs. With the implementation of strategies such as targeted poverty alleviation and rural revitalization, farmers' income has continued to grow and the urban-rural income gap has gradually narrowed [2]. It should be noted that although the implementation of the precise poverty alleviation strategy has eliminated absolute poverty under the current poverty standard, the situation of relatively backward rural economic development has not changed, and the problem of income inequality within rural areas remains prominent [3,4]. The income of rural residents has generally achieved sustained growth since the reform and opening up. However, in a relative sense, the income growth of rural low-income groups has been significantly slower. The average income level of the lowest 40% rural income group rose from 117 Yuan in 1980 to 7536 Yuan in 2020, while the overall level of rural per capita income rose from 191 Yuan to 17,132 Yuan in the same period. Judging from the income gap between different groups in rural areas, in 2020, the income difference between the top 20% of high-income households in China's rural areas and the lower 20% of low-income households in the same period will reach 8.23, and there is a trend of further expansion. How to promote the income growth of rural low-income groups and narrow the income gap within the countryside has become the key to solving the current problem of unbalanced and insufficient rural development, and is of great significance to promoting rural revitalization and achieving common prosperity.

For rural households, their income growth depends on the deepening of the agricultural division of labor and the effective employment of labor [5,6]. As an important manifestation of the deepening of the agricultural division of labor, agricultural mechanization services (AMS) have played an important role in improving agricultural production efficiency and facilitating labor transfer [7]. For example, Ma et al. [8] found that farmers are jointly making decisions to use farm machines and to work off the farm and that these two household activities affect maize yields and agrochemical expenses in different ways. Zhou et al. [9] showed that a 1% increase in farm machine use rate tends to decrease draft animal use by 2.82% in the long-run. Huan et al. [10] revealed that the effect of mechanization services on farm efficiency is significantly positive and is mediated by factor allocation.

The Chinese government has also responded positively to the development of AMS [11]. Since 2004, China started to implement the agricultural machinery purchase subsidy policy. In 2013, the Ministry of Agriculture issued the Opinions on Vigorously Promoting Agricultural Socialization Services, which pointed out the direction for the development of AMS. Subsequently, the annual No. 1 Central Document has made relevant deployments and requirements for the development of AMS. Under a series of policy support and institutional arrangements, the AMS market has developed rapidly. On the one hand, the degree of organization and specialization of the main body of AMS has been continuously improved. In 2020, the number of national agricultural mechanization service organizations reached 194,800, of which 30.39% of the service organizations have agricultural machinery worth then 500,000 Yuan and above. On the other hand, the market scale of AMS is expanding. The national revenue from AMS reached 478.15 billion Yuan in 2020, an increase of 97.46% over 2004.

As the service market continues to expand, more and more rural households use AMS for agricultural production [12,13]. So, whether or to what extent have AMS improved the income level of rural households in China? Several studies used the degree of change in the mean income to reflect the income effect of AMS and concluded that AMS positively affect the income of rural households [14,15]. However, due to differences in rural households’ capital endowments and factor allocations, the rate of return from the adoption of AMS by low-income rural households and high-income rural households may be significantly different. Will AMS widen or narrow the income gap of rural households? Existing research is still ambiguous. Due to the lack of a breakthrough in research methods, the research conclusions cannot accurately reflect the heterogeneous impact of AMS on different income groups, and there is also a lack of in-depth analysis of the relationship between AMS and rural household income gap. Therefore, it is necessary to deeply explore the relationship between AMS and the income level and income gap of rural households, so as to provide a new focus for improving the income inequality in rural areas and promoting the common prosperity of farmers and rural areas.

The objective of this study is to examine the impact of AMS on the income level and income gap of rural households. Compared with the existing literature, the possible marginal contributions of this paper mainly include the following three aspects: First, based on nationally representative micro-survey data, the unconditional quantile regression (UQR) method was used to compare the differences in the income effects of AMS on different income groups. It enriches the research on the relationship between AMS and the income of rural households. Second, the relationship between AMS and rural household income gap was investigated by using the recentered influence function (RIF) regression method. It provides new empirical evidence for accurately identifying the impact of AMS on the income gap of rural households. Third, from the perspective of regional differences and income source structure, the regional differences and structural effects of AMS on the income gap of rural households were explored. It provides new perspectives and references for formulating and implementing more effective policy measures.

## 2 Theoretical analysis and research hypotheses

### 2.1 Impact of AMS on the income of rural households

AMS can help rural households optimize resource allocation, thereby increasing their household income. Specifically, AMS have an impact on the income of rural households mainly through the following two aspects. On the one hand, AMS can accelerate labor transfer and increase off-farm employment time to boost rural households’ non-agricultural income growth [16]. AMS are an important manifestation of the deepening of the agricultural division of labor, which can effectively reduce the seasonal constraints of agricultural production on labor mobility. It has greatly liberated agricultural productivity and prompted them to go out to work and obtain more non-agricultural income from the heavy agricultural labor [17].

On the other hand, AMS can increase agricultural output and reduce production costs, thereby promoting agricultural income growth of rural households. As a typical labor-saving technological progress, agricultural mechanization can effectively improve labor productivity, reduce labor time and increase crop yield [18]. However, agricultural production by self-purchasing agricultural machinery often faces the problems of high investment threshold and easy locking of special assets [19]. The development of modern agricultural technology enables many agronomy and production links to be provided independently by professional social service organizations. Rural households replace direct investment by purchasing services in a roundabout way, which reduces the input of means of production and saves agricultural production costs [20]. Not only that, the AMS provider has advanced equipment and technology, and a higher degree of organization, which can effectively make up for the shortage of family labor and lack of technical experience. It can promote the scientific and green level of agricultural production, increase the output and effectively reduce the input of agricultural chemicals such as fertilizers and pesticides, thereby improving the efficiency of agricultural production [21].

### 2.2 Impact of AMS on the income gap of rural households

AMS can help narrow the differences in capital endowments among rural households, thereby reducing rural income inequality. In rural China, low-income rural households have very little physical capital [4]. A prominent manifestation is that there is a large gap between productive fixed assets represented by agricultural machinery and high-income rural households. In the case of an incomplete agricultural mechanization service market, as the relative factor prices of labor and capital rise, high-income rural households can purchase machinery to replace the scarce labor, so that the family labor resources can be allocated to the non-agricultural sector with higher returns [22]. Low-income rural households often face higher investment constraints and sunk costs when choosing whether to buy machinery [23].

The development and improvement of the agricultural mechanization service market can alleviate the financial and land constraints of low-income rural households and enable them to replace direct investment through the roundabout way of purchasing services. Usually, the small and subsistence farmers are not able to make investments in modern agricultural technologies such as water saving technologies for irrigation [[24], [25], [26]]. However, development of AMS effectively overcomes the contradiction between the small scale of land operation, weak purchasing ability and the demand for high horsepower machinery, and realizes the reasonable allocation of family labor between agricultural and non-agricultural industries [27]. The increase in non-agricultural working hours helps low-income rural households to increase their wage income, thereby narrowing the non-agricultural income gap with high-income rural households. At the same time, AMS not only replace labor input but also realize agricultural scale management through specialized division of labor and service aggregation. It helps low-income rural households to share the benefits of economies of scale, thereby narrowing the agricultural income gap with high-income rural households [28].

Based on the above analysis, this paper proposes the following research hypotheses: AMS can improve the income of rural households and reduce the income gap in rural areas.

## 3 Data, models, and variables

### 3.1 Data sources

The China Labor-force Dynamics Survey (CLDS) used a multi-stage, multi-level sampling method proportional to the size of the workforce. It has been launched in 28 provincial-level units across the country except Tibet, Xinjiang, Hainan, Hong Kong, Macao and Taiwan. The 2018 CLDS dataset included a total of 368 community questionnaires, 13,501 household questionnaires, and 16,537 individual questionnaires for the labor force population. There are two main reasons for choosing this database. First, as the national large-scale baseline survey data, it is representative and authoritative. Second, the data not only provides detailed information on household income and expenditure but also includes the degree of mechanization of grain production and the way of obtaining machinery. This paper firstly selected the households that were in rural areas as samples according to the research needs and then matched the household-level data with the village-level data. After excluding samples that did not engage in grain production and lacked key information, a sample of 3374 rural households were finally included in the analysis.

### 3.2 Model building

This paper uses the RIF regression method proposed by Firpo et al. [29] to test the relationship between AMS and the income level and income gap of rural households. Unlike OLS regression, which focuses on the effect of explanatory variables on the mean difference of the explained variables, RIF regression can reflect the marginal effects of explanatory variables on various distribution statistics of the explained variables. These statistics can be indicators such as mean, quantile, or Gini coefficient [30]. In addition, RIF regression is effective in weakening the endogeneity problem caused by omitted variables because it relaxes the independence assumption. The formula of RIF is as follows:

(1) $$RIF(y,v(F_{Y})=v(F_{Y})+IF(y,v(F_{Y}))$$

Among them, $v\left(F_{Y}\right)$ is the various statistics of y. $IF\left(y,v\left(F_{Y}\right)\right)$ is the influence function of y. Since the influence function has the characteristic of expectation equal to zero, the unconditional expectation of the RIF of the statistic is the statistic itself. Suppose the following linear relationship exists between $RIF\left(y,v\left(F_{Y}\right)\right)$ as the explained variable and the explanatory variable X: $RIF\left(y,v\left(F_{Y}\right)\right)=X^{'}\beta +\varepsilon_{i},E\left(\varepsilon_{i}\right)=0$. Taking unconditional expectations on both sides of the equation at the same time: $E\left[RIF\left(y,v\left(F_{Y}\right)\right)\right]=v\left(F_{Y}\right)=\bar{X^{\prime }}\beta$. β is the marginal effect of the marginal change in the explanatory variable X on the statistic $v\left(F_{Y}\right)$. When the distribution statistic is quantile, RIF regression is called UQR. Specifically for this study, in order to reveal the impact of AMS on the income of rural households at each quantile, the following models were constructed:

(2) $$RIF\left(\ln\;income_{i},q_{\tau }\right)=\alpha_{0}+\alpha_{1}AME_{i}+\alpha_{2}Control_{i}+\mu_{p}+\omega_{i}$$

Among them, $\ln\;income_{i}$ represents the logarithm of the total per capita income of rural households. $q_{\tau }$ represents different quartile levels. $AME_{i}$ represents the variable of AMS, which is measured by whether rural households use AMS in agricultural production; the coefficients $\alpha_{1}$ to be estimated measures the marginal effects of AMS on the income of rural households at different quantiles. $C{\text{ontrol}}_{i}$ is a series of control variables. $\mu_{p}$ is the province fixed effect to control the influence of many attribute differences between provinces. $\omega_{i}$ is random error term.

In order to further reveal the impact of AMS on the income gap of rural households, the following models were constructed:

(3) $$RIF\left(income_{i},v^{G\text{ini}}\right)=\lambda_{0}+\lambda_{1}AME_{i}+\lambda_{2}Control_{i}+\mu_{p}+\delta_{i}$$

Among them, $income_{i}$ represents the per capita annual income of rural households. $v^{G\text{ini}}$ represents Gini coefficient. The coefficients $\lambda_{1}$ to be estimated measures the marginal effect of AMS on the Gini coefficient of the total per capita income of rural households. $\delta_{i}$ is random error term.

### 3.3 Variable selection

Drawing on the practice of existing research [31], the logarithm of the total per capita income of rural households is used to reflect the income level of rural households, and the Gini coefficient of the total per capita income of rural households is used as an indicator to measure the income gap of rural households.

The core explanatory variable of this paper is AMS. It is necessary to explain the concept definition and variable measurement of AMS in detail. With the formation and development of the AMS market, scholars have conducted many discussions on the concept of AMS, but there is no clear and unified definition of the concept. In the existing research, AMS have many similar concepts, such as “agricultural machinery socialization service”, and “agricultural machinery operation service”. The differences between the concepts are mainly reflected in the two aspects of supply subject and service scope [32]. From the perspective of service scope, AMS in a broad sense not only include agricultural pre-production, mid-production, and post-production mechanical operation services but also relate to derivative services, such as agricultural machinery maintenance and technology promotion. The AMS in the narrow sense only refer to the mechanical operation service before, during, and after the production of agriculture. This paper studies AMS in a narrow sense, which specifically refers to the mechanical operation services provided by agricultural machinery service organizations and agricultural machinery households to agricultural producers. Based on the practice of existing research [33], the sample that purchased AMS was assigned a value of 1, and the sample that adopted traditional farming or all self-purchased agricultural machinery was assigned a value of 0.

Referring to relevant research [[34], [35], [36]], we mainly selected individual characteristic variables such as gender, age, education, party member, and health status, as well as family characteristic variables such as family labor ratio, off-farm labor ratio, land size, machine assets, government subsidies and gift expenditures as control variables. In addition, this paper also added dummy variables of provinces to control the effect at the regional level. The specific definitions and descriptive statistics of variables (province dummy variables are not listed) are shown in Table 1. In terms of the explanatory variables, the annual per capita household income of grain-growing rural households was generally low, with an average of 9851.72 Yuan. After calculation, it can be seen that the income ratio between the top 20% of high-income households and the bottom 20% of low-income households reached 26.19. There was a large income gap between high-income and low-income households. In terms of core explanatory variables, 51.45% of the rural households in the sample used AMS. This indicates that AMS have become more popular in agricultural production. In terms of control variables, the majority of household heads in the sample were male. The average age of household heads was about 56 years old, the average number of years of education was about 7 years, and the proportion of party members was only 7.71%. The mean value of physical health self-assessment was 3.45, which was between average to relatively healthy. The average share of the number of the labor force to total household size was 72.01% and the average share of the non-farm employed labor force to total household labor force was 34.98%. The per capita arable land area of households was 0.17 ha, the value of agricultural machinery owned averaged 2406.17 Yuan, the amount of government subsidies received averaged 633.47 Yuan, and the average gift expenditures of household for one year is 2494.54 Yuan.

**Table 1:** Variable definitions and descriptive statistics.

Variables	Definition	Mean	SD
Income	Per capita household income (yuan/capita)	9851.7204	13848.5306
AMS	1 if household adopted agricultural mechanization services, 0 otherwise	0.5145	0.4999
Gender	1 if household head is male, 0 otherwise	0.9129	0.2821
Age	Age of household head (years)	56.1666	11.3093
Education	Education of household head (years)	7.0765	3.2365
Party member	1 if household head is a party member, 0 otherwise	0.0771	0.2667
Health status	Self-reported health status of household head (from 1 = low to 5 = high)	3.4481	1.0400
Family labor ratio	Proportion of laborers to the number of family members	0.7201	0.2264
OFF-farm labor ratio	Proportion of off-farm laborers to the number of laborers	0.3498	0.2875
Land size	Per capita land size (hectares)	0.1712	0.3842
Machine assets	Monetary value of self-owned machine assets (yuan)	2406.1731	13619.1061
Government subsidies	Household receives cash subsidies for agricultural production (yuan)	633.4651	2239.1484
Gift expenditures	Household expenditures on social relations (yuan)	2494.5421	4364.8727

## 4 Empirical results

### 4.1 Impact of AMS on the income of rural households

In order to examine the distribution of the rate of return on the use of AMS among different income groups, this paper uses UQR to estimate the causal effect of AMS on the income of rural households at each quantile. UQR is a type of RIF regression, and this method can better deal with the influence of the distribution of control variables on the estimated results when estimating the quantile effect, and only consider the marginal influence of the core explanatory variables on the explained variables in the general sense. The results of UQR with 10, 25, 50, 75, and 90 as representative quantiles are reported in Table 2 and compared with the OLS regression results. In both UQR and OLS regressions, the explained variables are the logarithm of the annual per capita income of rural households.

**Table 2:** Impact of AMS on the income of rural households: UQR model estimation.

Variables	(1)	(2)	(3)	(4)	(5)	(6)
	10th	25th	50th	75th	90th	OLS
AMS	0.1619* (0.0826)	0.2728*** (0.0695)	0.2180*** (0.0536)	0.1711*** (0.0546)	0.1041* (0.0561)	0.1892*** (0.0413)
Constant	7.6425*** (0.4492)	8.9543*** (0.3656)	9.9104*** (0.2759)	9.4926*** (1.0688)	9.2518*** (0.2857)	9.0099*** (0.1785)
Control variables	Yes	Yes	Yes	Yes	Yes	Yes
Province dummy	Yes	Yes	Yes	Yes	Yes	Yes
Observations	3374	3374	3374	3374	3374	3374

Note: Standard errors appear in parentheses. ***, **, * indicate significant at the rejection of the null hypothesis at the 1%, 5%, and 10% significance levels, respectively.

The regression results show that AMS have a significant positive impact on the income of rural households at each quantile, but the estimated coefficients of AMS at different quantiles have significant differences. At the quantiles before the median, the UQR estimators are generally larger than the OLS estimators, while the UQR estimators at the quantiles after the median are generally smaller than the OLS estimators. This shows that AMS promote the income growth of rural households, but there are differences in the marginal effect on the household income of rural households at different quantiles. The rural households at the middle and low end of the income distribution obtain relatively greater benefits from AMS.

In the estimation of the impact of AMS on the income of rural households, the factors that affect both the use of AMS and the income of rural households were controlled in the model as much as possible, but some unobservable variables, such as the personal ability and risk appetite of rural households. These factors were left out in the errors, leading to biased and inconsistent estimates. In order to alleviate the endogeneity problem, this paper used an instrumental variable approach to re-estimate the model. Referring to existing research [37], the average level of AMS of other rural households in the village was selected as an instrumental variable. Its rationality lies in the fact that the agricultural production decision-making of rural households in China has a group effect, and the decision-making of AMS of rural households will be affected by other rural households in the village, and the use of AMS of other rural households in the village does not directly affect the income of rural households, which meets the correlation and exogenous requirements of instrumental variable selection theoretically.

Table 3 shows the estimation results based on instrumental variables. The F-test statistic value of instrumental variables in the first stage is much larger than the empirical value of 10, and there is no weak instrumental variable problem, which further proves that the selection of instrumental variables is effective. The regression results in the second stage show that after eliminating the endogeneity problem, the estimated coefficients of AMS are all significantly positive, and show a trend of greater impact on low-income and middle-income rural households and less impact on high-income rural households, which is consistent with the previous estimation results.

**Table 3:** Impact of AMS on the income of rural households: Instrumental variable approach estimation.

Variables	(1)	(2)	(3)	(4)	(5)	(6)
	10th	25th	50th	75th	90th	IV-OLS
AMS	0.3822** (0.1669)	0.5042*** (0.1276)	0.4390*** (0.0974)	0.3025*** (0.0977)	0.2287** (0.1033)	0.3997*** (0.0766)
Constant	7.5243*** (0.4060)	8.8302*** (0.3236)	9.9188*** (0.2337)	9.4221*** (1.1102)	9.1850*** (0.2577)	8.8970*** (0.2137)
Control variables	Yes	Yes	Yes	Yes	Yes	Yes
Province dummy	Yes	Yes	Yes	Yes	Yes	Yes
Observations	3374	3374	3374	3374	3374	3374

Note: Standard errors appear in parentheses. *** and ** indicate significant at the rejection of the null hypothesis at the 1% and 5% significance levels, respectively.

### 4.2 Impact of AMS on the income gap of rural households

Table 4 reports the results of the RIF estimation of the income gap of rural households by AMS. The rural household income gap is measured in the model in three forms.

**Table 4:** Impact of AMS on income gap of rural households.

Variables	(1)	(2)	(3)
	Gini coefficient	90th-50th quantiles	50th-10th quantiles
AMS	−0.0519** (0.0220)	−0.1139* (0.0656)	0.0561 (0.0874)
Constant	0.3773*** (0.0856)	−0.6586*** (0.2385)	2.2680*** (0.3698)
Control variables	Yes	Yes	Yes
Province dummy	Yes	Yes	Yes
Observations	3374	3374	3374

Note: Standard errors appear in parentheses. ***, ** and * indicate significant at the rejection of the null hypothesis at the 1%, 5%, and 10% significance levels, respectively.

Specifically, the Gini coefficient reflects the degree of inequality in the overall income distribution. 90-50th percentile log income difference and 50-10th percentile log income difference measure inequality at the high end of the income distribution and inequality at the low end, respectively. Column (1) is the RIF regression result based on the Gini coefficient. It can be seen that the estimated coefficient of AMS is −0.0548, which is significant at the 1% level. This shows that AMS are “pro-poor” and help to reduce the income gap among rural households while their income is increased. Columns (2) and (3) are the RIF regression results based on the 90-50th quantile log income difference and the 50-10th quantile log income difference, respectively. The results show that the estimated coefficient of AMS is significantly negative in column (2) but not significant in column (3). It can be seen that, compared with narrowing the income gap between middle-income and low-income rural households, AMS have a more significant effect on narrowing the income gap between high-income and middle-income rural households.

### 4.3 Robustness test

This paper used a series of tests such as the instrumental variable approach, replacing explanatory variables, and changing income gap measurement indicators to verify the robustness of the conclusions. Table 5 reports the robustness test results. Column (1) in Table 5 is the RIF estimation results using the instrumental variable approach. The regression results in the second stage show that the coefficient of the AMS is −0.0882, and it is significant at the 1% level, indicating that the AMS have played a role in reducing the income gaps between rural households. Compared with the model estimation results without instrumental variables, the estimated coefficients of key explanatory variables did not change in significance level and direction, proving the robustness of the benchmark regression conclusions.

**Table 5:** Impact of AMS on income gap of rural households: robustness test.

Variables	(1)	(2)	(3)	(4)	(5)
	IV-RIF	Replacement for explanatory variables	Generalized entropy index		Atkinson index
			α = 0	α = 1	ε = 1
AMS	−0.0882*** (0.0297)		−0.1094** (0.0475)	−0.1506* (0.0822)	−0.0593** (0.0258)
Service use intensity		−0.0338** (0.0138)			
Constant	−0.3968*** (0.0923)	0.4028*** (0.0871)	0.2177 (0.1619)	0.2972 (0.3116)	0.2439** (0.0878)
Control variables	Yes	Yes	Yes	Yes	Yes
Province dummy	Yes	Yes	Yes	Yes	Yes
Observations	3374	3374	3374	3374	3374

Note: Standard errors appear in parentheses. ***, ** and * indicate significant at the rejection of the null hypothesis at the 1%, 5%, and 10% significance levels, respectively.

The key explanatory variable is whether rural households use AMS in this paper. In order to examine the robustness of the estimated results, further use of service usage to measure, and re-estimate the impact of AMS on the income gap of rural households. This paper drew on the practice of Ma et al. [38] to define the degree of service usage. Specifically, if rural households do not use AMS, the value is 1, if they partly used, the value is 2, and if they used in all production stages, the value is 3. The regression results in column (2) of Table 5 show that the coefficient of service usage is −0.0338, which is significant at the 5% level, indicating that the research conclusions of this paper will not be affected by the key explanatory variables.

In addition to the Gini coefficient, the Generalized Entropy index and the Atkinson index can take into account different forms of income distribution and are also widely used indicators to measure inequality [39]. Therefore, this paper used the Generalized Entropy index and the Atkinson index as supplementary indicators of the income gap for RIF regression. Among them, the Generalized Entropy index is related to the value of α, the smaller value of α is more sensitive to the lower tail of the income distribution, the larger value of α is more sensitive to the upper tail of the income distribution and the value of α is generally 0 and 1. The Atkinson index also contains ε that represents the aversion degree of inequality, generally taking a value of 1. The regression results of columns (3) to (5) in Table 5 show that the estimated coefficients of AMS are all significantly negative, indicating that AMS can narrow the income gap of rural households. The negative impact of AMS on the income gap of rural households does not change due to the change of the measures of the income gap, indicating that the conclusions of this paper are relatively stable.

### 4.4 Further analysis based on regional heterogeneity

In China, huge differences in economic and social development exist in different regions, the problem of unbalanced rural development is particularly prominent, and the impact of AMS on the income gap of rural households is likely to have regional heterogeneity. Due to the superior geographical location and the support of national policies, the economic development level and marketization degree of the eastern coastal areas are much higher than those of the central and western regions in the same period, and there are also obvious differences in the resource endowment and income status of rural residents. For this reason, this paper divides the country into three major regional types, namely, the eastern region, the central region and the western region, and conducts IV-RIF regression on each regional sample separately.^1^

The estimation results in column (1) of Table 6 show that the impact of AMS on the income gap of rural households is significantly negative in the eastern and western regions with coefficients of −0.1449 and −0.1152 for the AMS variable, respectively. In contrast, in the central region, the impact of AMS on the income gap of rural households was not significant. The above results indicate that there are regional differences in the impact of AMS on the income gap of rural households, and the impact of AMS in reducing the income gap of rural households is more obvious in the eastern and western regions. This may be mainly due to the higher level of industrialization and abundant non-farm employment opportunities in the eastern region. This facilitates the transfer of labor replaced by AMS to the non-farm sector and promotes the upgrading of income levels of low-income households. In addition, the level of agricultural mechanization in the western region still has more room for improvement. AMS effectively lower the threshold for low-income farmers to adopt mechanized production, which in turn alleviates the income inequality due to differences in capital endowment.

**Table 6:** Impact of AMS on the income gap of rural households in different regions.

Variables	(1)	(2)	(3)
	Eastern	Central	Western
AMS	−0.1449* (0.0758)	0.0056 (0.0323)	−0.1152*** (0.0360)
Constant	0.5206*** (0.1640)	0.4276*** (0.0828)	0.7682*** (0.1713)
Control variables	Yes	Yes	Yes
Province dummy	Yes	Yes	Yes
Observations	1151	1082	1141

Note: Standard errors appear in parentheses. *** and * indicate significant at the rejection of the null hypothesis at the 1% and 10% significance levels, respectively.

### 4.5 Further analysis based on the structure of income sources

Benchmark regression results confirm that AMS significantly reduce the income gap of rural households, but the effect on different types of the income gap is still ambiguous. Further analysis of the income source structure of rural households will help to clarify the internal mechanism of the impact of AMS on the income gap of rural households. In view of this, the per capita income of rural households was decomposed with reference to existing studies [40], and the different effects of AMS on the agricultural income gap and the non-agricultural income gap of rural households were explored separately. Agricultural income includes planting income, forestry income, livestock income, and fishery income. Non-agricultural income includes non-agricultural business income and the income of wages and salaries.

The estimation results in Table 7 show that AMS have a negative impact on the agricultural income gap and non-agricultural income gap of rural households, but there are differences in the estimated coefficients and significance. From the regression results in column (1) of Table 7, it can be seen that the estimated coefficient of AMS is negative but not significant, indicating that the role of AMS in reducing the agricultural income gap is not obvious. Column (2) of Table 7 shows the estimated results of the impact of AMS on the non-agricultural income gap, the estimated coefficient of AMS is −0.0563 and is significant at the 5% level. The above estimates show that AMS alleviate overall income inequality mainly by narrowing the non-agricultural income gap of rural households. A reasonable explanation is that low-income rural households realize intensive cultivation of land through “over-intensification” of labor, and there is no comparative disadvantage in terms of land productivity. At the same time, agricultural products such as grain, as a necessity of social production and life, have a small price elasticity of demand. The total demand remains unchanged, and the increase in production does not bring a significant increase in income. The main source of income growth for rural households is the wages and salaries of migrant workers. The development of the AMS market has created conditions for capital and technology to replace labor. By purchasing AMS, low-income groups promote the transfer of household labor to the non-agricultural sector while taking agricultural production into account, thereby narrowing the non-agricultural income gap with high-income rural households.

**Table 7:** Impact of AMS on the income gap of different types of rural households.

Variables	(1)	(2)
	Agricultural income gap	Non-agricultural income gap
AMS	−0.0167 (0.0277)	−0.0563** (0.0262)
Constant	1.1285*** (0.0502)	0.6436*** (0.2315)
Control variables	Yes	Yes
Province dummy	Yes	Yes
Observations	3374	3374

Note: Standard errors appear in parentheses. *** and ** indicate significant at the rejection of the null hypothesis at the 1% and 5% significance levels, respectively.

## 5 Discussion

Using the recentered influence function regression method and publicly available data from the China Labor-force Dynamics Survey, this paper investigates the impact of agricultural mechanization services (AMS) on rural household income and income gap. The results of this study have some similarities and differences with the conclusions of other AMS studies. For example, Lu et al. [7] found that switching to AMS for plowing, transplanting and harvesting can improve rice yield and increase farmers’ income. Tang et al. [20] showed that AMS can help improve cost efficiency, thus contributing to reduce production costs and increase the income of farmers. As in their studies, we reveal that AMS have significantly increased rural household income. In contrast to their studies, our findings show that the income effect of AMS is not homogenous. The effect of income growth on medium-income and low-income groups is greater. This study indicates that AMS help to narrow the income gap between rural households. This in line with the finding of Zhou et al. [41], who also found that the low-productive farmers tend to benefit more from farm machinery use relative to their high-productive counterparts, and farm machinery use reduces the inequality of maize yields. We find that the effect of AMS on reducing the income gap between rural households in the eastern and western regions is significantly stronger than that in the central region. Our results are similar to the findings of Mi et al. [15], who reported that the differences in the resource endowments of southern and northern Xinjiang result in significant regional differences in the welfare effects of AMS. This study reveals that AMS can significantly reduce the non-agricultural income gap of rural households, but the impact on the agricultural income gap is negligible. This observation is consistent with Qian et al. [32], who confirmed that AMS can reduce the labor burden of rural households and promote off-farm employment of some family members.

## 6 Conclusions, policy implications and future research

### 6.1 Research conclusions

AMS are an important link between rural households and modern agriculture, whether its rapid development contributes to the common prosperity of rural areas is an urgent problem to be studied. Based on the 2018 CLDS data, this paper used the RIF regression method to explore the relationship between AMS and the income and income gap of rural households. The paper found that: First, AMS positively affect the income of rural households in different quantiles, but there are significant differences in the marginal effect on the income of rural households in different quantiles. Second, AMS have significantly narrowed the overall income gap of rural households. After using an instrumental variable approach, replacing the explanatory variables, and changing the income gap measurement indicators, the research conclusion is still stable. From the perspective of the internal structure of the income gap, agricultural AMS play an important role in narrowing the gap at the high end of the income distribution but have little effect on the gap at the low end of the income distribution. Third, Further analysis based on regional heterogeneity found that AMS contribute to the reduction of rural household income gap in the eastern and western regions, but the impact on rural household income gap in the central region is not significant. Finally, from the perspective of income source structure, AMS help to narrow the non-agricultural income gap, but the role in narrowing the agricultural income gap is not obvious.

### 6.2 Policy implications

Our findings have practical implications for promoting rural revitalization and achieve common prosperity. Given the significance of AMS in increasing the income of rural households and alleviating the income gap between rural households, policies should be directed towards reducing the constraints that hinder the AMS adoption in rural regions. On the one hand, the government should continue to strengthen the construction of the agricultural socialized service system and provide favorable policy conditions for improving the breadth and depth of services. Continuously improve the quality of AMS supply and the level of technology, support service organizations with strong operational capabilities to provide rural households with various forms of high-quality service projects, and create favorable conditions for meeting and expanding the service needs of rural households. On the other hand, the government should consider subsidizing the purchase of AMS for low-income groups to alleviate the financial constraints they face in adopting AMS. This will guide more low-income rural households to participate in non-farm employment and narrow the non-agricultural income gap with high-income rural households.

### 6.3 Limitations of the study and future research

This study focuses on responding to the question of whether AMS can help narrow the income gap of rural households, and provides new empirical evidence for a comprehensive assessment of the economic and social impacts of AMS development. However, it should be pointed out that there are still some shortcomings in this study that need to be further expanded in future research. First, this study only uses the 2018 CLDS data to examine the relationship between AMS and rural households’ income and income gap, and there is still a problem of insufficient sample size. In the future, more representative large-scale random sample data should be used to verify the conclusions drawn in this paper. Second, due to the lack of data information, this paper does not consider the differences in the impact of AMS in different production links on the income and income gap of rural households. In-depth analysis can be carried out in the follow-up research. Third, given that studies investigating the nexus between AMS and the income gap of rural households remain scarce, further exploration is needed to clarify the underlying mechanisms through which AMS operates on the income gap of rural households.

## Author contribution statement

Xiance Sang, PhD Scholar: Conceived and designed the experiments; Performed the experiments; Analyzed and interpreted the data; Wrote the paper.

Xiaofeng Luo, PhD: Analyzed and interpreted the data; Wrote the paper.

Amar Razzaq, PhD: Contributed reagents, materials, analysis tools or data; Wrote the paper.

Yanzhong Huang; Sahar Erfanian, PhD: Contributed reagents, materials, analysis tools or data.

## Funding statement

Xiaofeng Luo was supported by National Natural Science Foundation of China [72073048], Key Program of the 10.13039/501100012456National Social Science Foundation of China [20AZD091].

## Data availability statement

Data will be made available on request.

## Additional information

No additional information is available for this paper.

## Footnotes

1 Among the 26 provinces covered by the sample rural households, 9 provinces involving Beijing, Tianjin, Hebei, Liaoning, Jiangsu, Zhejiang, Fujian, Shandong, and Guangdong belong to the eastern region; 8 provinces involving Shanxi, Jilin, Heilongjiang, Anhui, Jiangxi, Henan, Hubei, and Hunan belong to the central region; 9 provinces involving Sichuan, Chongqing, Guizhou, Yunnan, Shaanxi, Gansu, Ningxia, Guangxi, and Inner Mongolia belong to the western region.
